# Peripheral neuropathy in the pre-diabetic state of the type 2 diabetes mouse model (TSOD mice) involves TRPV1 expression in dorsal root ganglions

**DOI:** 10.1016/j.ibneur.2022.02.001

**Published:** 2022-02-08

**Authors:** Kyoko Shida, Masahiro Ohsawa, Satoru Takahashi, Haruko Ota, Tetsuya Tamura, Nobuyoshi Kusama, Mina Nakasone, Hisaaki Yamazaki, Kazuya Sobue

**Affiliations:** a Department of Anesthesiology and Intensive Care Medicine, Nagoya City University Graduate School of Medical Sciences, Nagoya, Japan; b Laboratory of CNS Pharmacology, Nagoya City University Graduate School of Pharmaceutical Sciences, Nagoya, Japan; c Department of Experimental Pathology and Tumor Biology, Nagoya City University Graduate School of Medical Sciences, Nagoya, Japan

**Keywords:** DM, diabetes mellitus, IGT, impaired glucose tolerance, TSOD, Tsumura Suzuki Obese Diabetes, TSNO, Tsumura Suzuki Non-Obesity, ITT, insulin tolerance test, DRG, dorsal root ganglion, TRPV1, Transient Receptor Potential Vanilloid 1, NF-H, neurofilament heavy, FITC, fluorescein isothiocyanate, TRITC, tetramethylrhodamine, ANOVA, Analysis of variance, ir, immunoreactive, tHODE, total Hydroxyoctadecadienoic Acid, LPA, Lysophosphatidic Acid, STZ, streptozotocin, HFD, High-Fat Diet, Diabetes mellitus, Impaired glucose tolerance, Mechanical allodynia, Thermal hypersensitivity

## Abstract

Peripheral neuropathy, which is a complication of diabetes mellitus (DM), is thought to occur in the pre-DM state, being known as impaired glucose tolerance (IGT) neuropathy, although its pathogenesis is unknown. Since it is reversible, an effective treatment at the pre-DM stage could stop the progression of peripheral neuropathy and improve patients’ quality of life and reduce medical costs. We investigated the hypersensitivity to mechanical and thermal stimuli during the pre-DM state in Tsumura Suzuki Obese Diabetes (TSOD) mice, a type 2 DM mouse model. The expression pattern of the Transient Receptor Potential Vanilloid 1 (TRPV1)-positive cells in the dorsal root ganglia (DRG) was examined in TSOD mice, which showed a pre-DM state at 5–12 weeks of age and decreased mechanical and thermal nociceptive thresholds. Additionally, the size of TRPV1-positive cells in TSOD mice increased compared with that in non-diabetic controls (Tsumura Suzuki Non-Obesity; TSNO). Furthermore, the expression of TRPV1 on myelinated nerve fibers (neurofilament heavy-positive cells) had significantly increased. Thus, TSOD mice in the pre-DM state at 5–12 weeks of age could be a useful animal model of IGT neuropathy. We also hypothesized that the development of IGT neuropathy may involve a switch in TRPV1 expression from small, unmyelinated neurons to large, myelinated neurons in the DRG.

## Highlights

• The mechanism of peripheral neuropathy was investigated in insulin resistance state.
• Tsumura Suzuki Obese Diabetes (TSOD) mice showed a pre-diabetic state.
• TSOD mice showed mechanical and thermal hypersensitivity in a pre-diabetic state.
• The TRPV1 expression shifted to larger dorsal root ganglia (DRG) cells in TSOD mice.
• TRPV1 expression in myelinated neurons of the DRG was involved in neuropathy.

## 1 Introduction

The number of people with diabetes mellitus (DM) worldwide is on the rise, estimated at 463 million in 2019, according to the latest data (Saeedi et al., 2019). DM neuropathy is one of the most common microvascular complications of DM, and The Rochester Diabetic Neuropathy Study reported that the prevalence of neuropathy in type 2 DM would be 45.0% (Dyck et al., 1993). Recent reports also showed that approximately 50% of adult DM patients would be affected by peripheral neuropathy in their lifetime (Hicks and Selvin, 2019).

The pathogenesis of DM neuropathy is still unknown despite many studies. However, hyperglycemia plays a critical role in DM neuropathy progression. It is hypothesized that reactive oxygen species resulting from a combination of metabolic dysfunction, inflammatory responses, and mitochondrial dysfunction caused by persistent hyperglycemia may cause protein modification, lipid peroxidation, and DNA damage, ultimately leading to neurological dysfunction from demyelination and axonal degeneration (Carrasco et al., 2018, Román-Pintos et al., 2016, Yagihashi et al., 2011).

Peripheral neuropathy in DM has been reported to occur in the pre-DM state, being known as impaired glucose tolerance (IGT) neuropathy (Sumner et al., 2003), while its pathogenic mechanism remains unknown. Pre-DM status is the earliest stage of glucose intolerance and appears before the onset of type 2 diabetes. The American Diabetes Association defines pre-DM status as HbA1c = 5.7–6.4% (Stino and Smith, 2017).

DM peripheral neuropathy becomes irreversible. However, IGT neuropathy in the pre-DM state would be reversible (Hinder et al., 2017). Because peripheral neuropathy has a significant impact on patients’ quality of life and healthcare costs, addressing IGT neuropathy in a reversible phase may help avoid these problems. Therefore, there is an urgent need to elucidate the pathogenesis and treatment of IGT neuropathy. However, there are a few studies on it using animal models.

Tsumura Suzuki Obese Diabetes (TSOD) mice are a polygenic type 2 DM model derived from ddY mice, while Tsumura Suzuki Non-Obesity (TSNO) mice are used as non-diabetic controls. TSOD mice show visceral fat obesity, similar to Asian obesity, and present with hyperglycemia, hyperinsulinemia, IGT, and urinary glucose, with symptoms similar to human complications of DM (renal impairment, fatty liver, and peripheral neuropathy) (Hirayama et al., 1999). In a previous study, TSOD mice showed DM symptoms after 11 weeks of age (Murotomi et al., 2014), and during the rearing period of TSOD mice, blood glucose levels did not increase until 12 weeks of age and the urine glucose positivity rate was low.

In this study, we assumed that the pre-DM state in TSOD mice is up to 12 weeks of age. Then, we confirmed the symptoms of IGT neuropathy in the pre-DM state of TSOD mice and investigated its pathogenesis.

## 2 Experimental procedures

### 2.1 Animals

Male TSOD mice (5–7 weeks old, 30–40 g body weight) and male TSNO mice (5–7 weeks old, 20–27 g body weight) were obtained from the Institute for Animal Reproduction in Ibaraki, Japan. Six mice each were placed in a cage and kept at 23 ± 2 °C with 12 h light/dark cycles. Food (MFG, Oriental Yeast Co., Ltd, Tokyo, Japan) and water were consumed ad libitum. All experiments were conducted in accordance with the National Institute of Health Guide for the Care and Use of Laboratory Animals (NIH Publications No. 80–23) revised 1996 and conducted with the approval of the Animal Experiment Committee of the Nagoya City University Graduate School of Medicine (No. M29M-24).

### 2.2 Confirmation of mouse conditions

Body weight and blood and urine glucose levels were measured weekly (urine glucose only for TSOD, starting from 9 weeks of age). Blood glucose levels were measured by a glucose analyzer, StatStripXP2 (Nipro Corporation, Osaka, Japan). To collect blood samples, mice were placed in a prone beaker, and underwent a small incision at the tip of the tail with a surgical scalpel. The drained blood (about 2 μL) was pooled. Urine glucose was measured by holding the mouse by hand and massaging the lower abdomen and soaking New Uriace Ga (Terumo Corporation, Tokyo, Japan) test paper in the urine released by the mouse.

The insulin tolerance test (ITT) was performed after 3 h of fasting. Insulin (0.5 Units/kg; Humalog, Eli Lilly Japan, Kobe, Japan) was administered intraperitoneally. Blood glucose levels were measured at 0, 30, 60, and 90 min after insulin administration.

### 2.3 Assessment of mechanical allodynia

Nociception was measured using Dixon’s up-down method (Dixon, 1980). That is, each mouse was placed in a clear glass cup on a metal net, and the mechanical nociceptive threshold was measured using von Frey filaments (Touch-Test, North Coast Medical Inc., CA, USA). Foot retraction of the likelihood of a response occurring at 50% (50% threshold) was determined. Eight von Frey filaments (0.02, 0.04, 0.07, 0.16, 0.4, 0.6, 1.0, and 1.4 g) were selected. The test was started with a 0.16 g filament and the plantar surface of the hind foot was pressed vertically for 3–4 s with enough force to bend the filament. If the foot retracted, it was a positive response, and the next weaker (i.e., lighter) filament was then used. If there was no foot retraction response, the next stronger (i.e., heavier) filament was used. This procedure was continued until 4 measurements were obtained after a positive reaction or until 4 consecutive positive or 5 negative reactions were obtained, and the results obtained were used to calculate the 50% threshold.

### 2.4 Assessment of thermal hypersensitivity

The hot plate device (HOT PLATE ANALGESIA METER MK-350 C, Muromachi Kikai Co., Ltd, Tokyo, Japan) consists of an electrically heated plate surface and is equipped with a temperature control device that maintains the temperature at a constant 51 °C (Elmer et al., 1998). In the hot plate test, the latency to the nociceptive response (licking or jumping) was measured as an index of nociceptive perception. The cut-off time was set at 60 s to avoid tissue damage.

The latency of the nociceptive response was determined by averaging the 3 measurements made 5 times per mouse, excluding the maximum and minimum values, and was compared across 6- and 12-week-old TSNO and TSOD mice (n = 6).

### 2.5 Immunohistochemistry

Twelve-week-old TSOD and TSNO mice (n = 5) were anesthetized with 2% isoflurane and bled to death. The spinal canal was first cut out, the spinal cord was then removed, and the dorsal root ganglion (DRG) of L4, L5, and L6 was harvested.

The DRG were immersed in 10% neutral buffered formalin for 15 h at room temperature. Anti-TRPV1 (Transient Receptor Potential Vanilloid 1) antibody (Alomone labs, ACC-030, 1:5000) was used to detect the DRG. Immunostaining of TRPV1 was performed using BOND-MAX (Leica Biosystems, Wetzlar, Germany) according to the manufacturer’s instructions.

For diameter distribution analysis of TRPV1-positive cells in DRG, ImageJ was used to measure the diameter of brown TRPV1-positive cells (Light et al., 2008, Price and Flores, 2007). After approximating the positive cells to an ellipse, the radius of the approximate circle was calculated and used as the positive cell diameter. Additionally, a histogram of TRPV1-positive cell diameter was generated.

Immunofluorescence double staining for TRPV1 and neurofilament heavy (NF-H) was also performed using the same DRG samples as above. After deparaffinization, the samples were blocked with Histofine Mouse Stain Kit (Nichirei Bioscience, Tokyo, Japan) and stained with anti-TRPV1 antibody (Alomone labs, ACC-030, 1:1000) and anti-NF-H antibody (Cell Signaling Technologies, #2836, 1:200). Then, as secondary antibodies, fluorescein isothiocyanate (FITC) goat anti-rabbit IgG (Invitrogen, F2765, 1:100) was used for TRPV1 and tetramethylrhodamine (TRITC) goat anti-mouse IgG (Invitrogen, T2762, 1:100) for NF-H. For autofluorescence suppression, the Vector TrueVIEW Autofluorescence Quenching Kit (Vector Laboratories, CA, USA) was used. Then, the slides were sealed in VECTASHIELD Mounting Medium (Vector Laboratories, CA, USA) and cell nuclei were stained with DAPI. The immunolabeling fluorescence was detected using a confocal microscope (LSM 800, Zen software, Carl Zeiss AG).

### 2.6 Statistical analysis

Data are expressed as mean ± SEM, and differences between the 2 groups were analyzed, being first assessed by the F-test, using the Student’s t-test for normal distributions and the Welch’s t-test for non-normal distributions. Sigma Plot (Hulinks Inc., Tokyo, Japan) was used for the statistics of differences between multiple groups, which were evaluated by the Tukey test after a two-way ANOVA. Differences in probability values of less than 0.05 (P < 0.05) were considered statistically significant.

## 3 Results

### 3.1 TSOD mice were heavier and had higher blood glucose levels

Body weight and non-fasting blood glucose levels were measured in TSOD and TSNO mice from 6 to 16 weeks of age. Urinary glucose was checked in TSOD mice only at the age of 9–16 weeks.

**Fig. 1:**
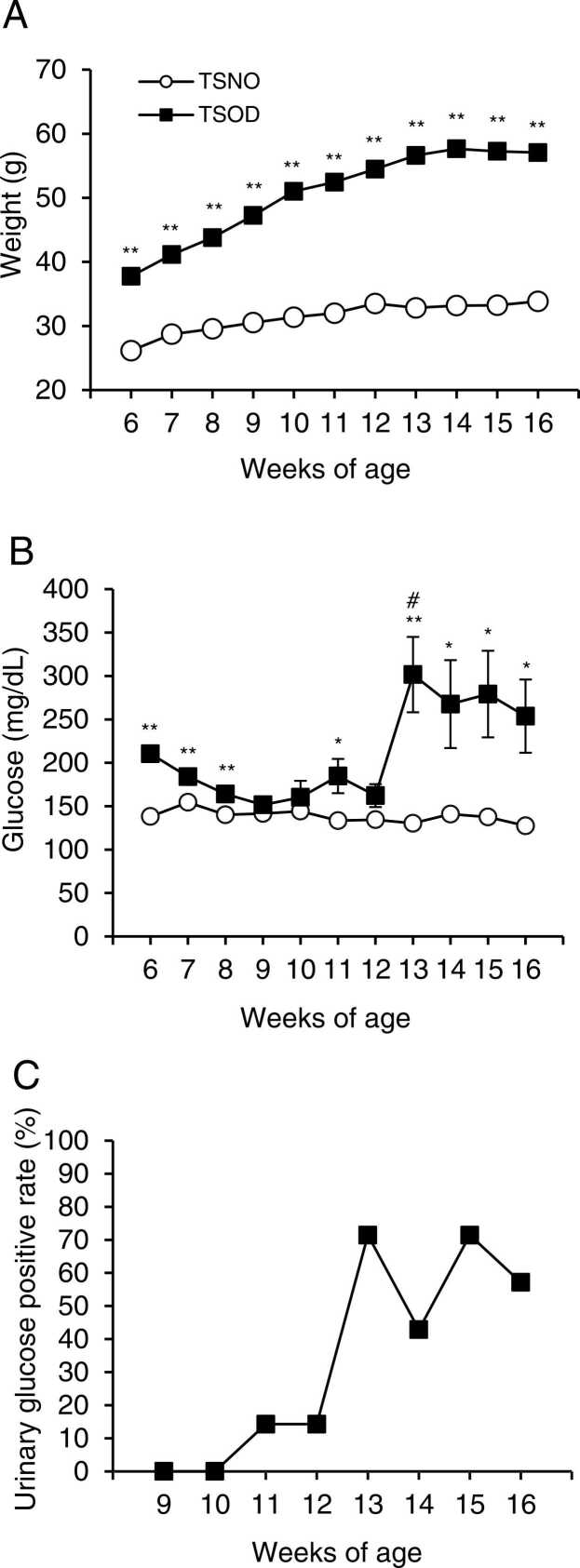
Changes in body weight (A), blood glucose levels (B), and urine glucose positivity rate (C, only TSOD mice) in TSOD and TSNO mice. Blood glucose levels were measured using a StatStripXp2 glucose analyzer by incising the tip of the tail. Urinary glucose was measured using New Uriace Ga test papers from 7 mice, and the positive rate is shown in the graph (C). Each point (A and B) represents the mean ± S.E.M. of 7 mice. Significant differences between groups were determined by a t-test. If no error bar was visible, the value was smaller than the symbol. *P < 0.05, **P < 0.01; TSNO mice (open circle) vs. TSOD mice (closed square) (A and B). #P < 0.05; comparison of non-fasting blood glucose levels of TSOD mice in 12 weeks vs. 13 weeks in TSOD mice (B).

The mean body weight was significantly heavier in TSOD mice at all ages, with a mean body weight of 33.8 ± 0.7 g in TSNO mice and 57.1 ± 1.1 g in TSOD mice at 16 weeks (P < 0.01), (Fig. 1A). Non-fasting blood glucose levels did not increase and did not exceed 250 mg/dL in TSOD mice until 12 weeks of age, but they averaged over 250 mg/dL after 13 weeks of age. The mean value of non-fasting blood glucose levels in 13-week-old TSNO mice was 130 ± 7 mg/dL, while that in TSOD mice was as high as 302 ± 43 mg/dL, which was significantly higher than that in TSNO mice (P < 0.05). The mean non-fasting blood glucose levels of TSOD mice were significantly different between 12 weeks of age (162 ± 13 mg/dL) and 13 weeks of age (302 ± 43 mg/dL) (P < 0.05, Fig. 1B). Furthermore, the positive rate of urinary glucose in TSOD mice up to 12 weeks of age was 14.3%, but at 13–16 weeks of age, it ranged from 42.9% to 71.4% (Fig. 1C).

### 3.2 TSOD mice were insulin-resistant after 5 weeks of age

To investigate insulin resistance in TSNO and TSOD mice, the ITT was performed on 5-week-old mice. TSNO and TSOD mice were divided into saline (control) and insulin groups, respectively, for 4 groups (n = 5).

**Fig. 2:**
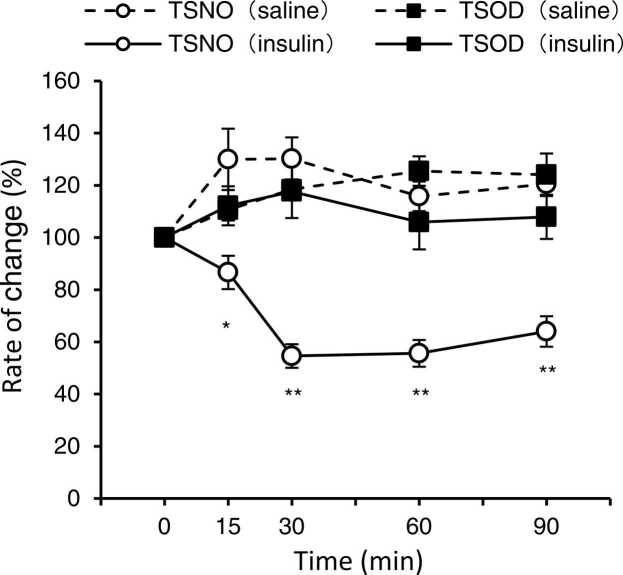
Effect of insulin on the serum glucose levels in TSOD and TSNO mice. Five-week-old mice fasted for 3 h, and 0.5 Units/kg of insulin (control group: saline) were administered intraperitoneally. Blood glucose levels were measured at 0, 30, 60, and 90 min after administration, and the percent change in blood glucose levels was indicated. Each point represents the mean ± S.E.M. of 5 mice. Significant differences between groups were determined by a t-test. If no error bar was visible, the value was smaller than the symbol. The TSNO mice saline group is indicated by an open circle with a broken line, and the TSOD mice saline group is indicated by closed square with a broken line. *P < 0.05, **P < 0.01; insulin-treated TSNO mice group (open circles with solid line) vs. insulin-treated TSOD mice group (closed square with solid line).

In the saline group, no changes in blood glucose levels were observed. In the insulin group of TSNO mice, blood glucose levels decreased from 15 min after administration and continued to do so until 90 min after administration. In the insulin group of TSOD mice, there was little change in blood glucose levels. Blood glucose levels in the insulin-treated group were significantly lower in TSNO mice than those in TSOD mice (P < 0.05, Fig. 2).

Based on the above, we defined the pre-DM state of TSOD mice as the period from 5 to 12 weeks of age, when the average non-fasting blood glucose level was less than 250 mL/dL and the urine glucose positivity rate was less than 40%.

### 3.3 Mechanical nociception was decreased in TSOD mice in the pre-DM state

**Fig. 3:**
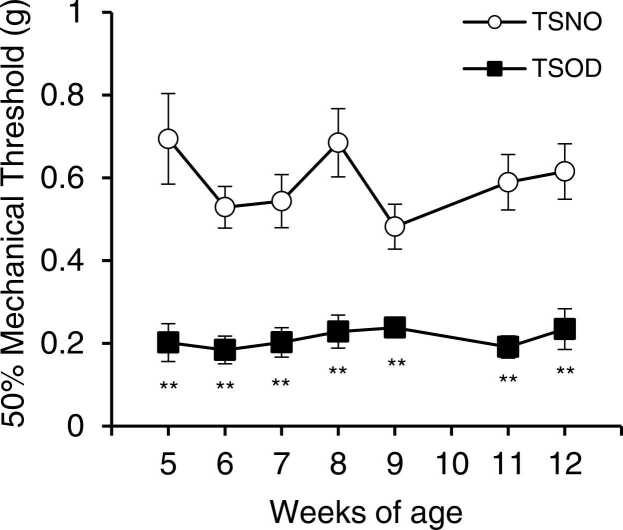
Time-course of mechanical nociceptive threshold in TSOD and TSNO mice. The von Frey test was performed on 5–12 weeks TSNO and TSOD mice to evaluate the change in mechanical nociceptive threshold. Each point represents the mean ± S.E.M. of 6 mice. Significant differences between groups were determined by a t-test. If the error bars are not visible, the value was smaller than the symbol. **P < 0.01; TSNO mice (open circle) vs. TSOD mice (closed square).

To evaluate mechanical nociceptive thresholds in TSNO and pre-DM state TSOD mice, von Frey tests were conducted at 5–12 weeks of age (n = 6). The 50% mechanical threshold (%) remained around 0.6 g in TSNO mice, but around 0.2 g in TSOD mice. A significant decrease in the mechanical nociceptive threshold was observed in TSOD mice aged 5–12 weeks (p < 0.01, Fig. 3).

### 3.4 Hypersensitivity to thermal stimuli was increased in TSOD mice in the pre-DM state

**Fig. 4:**
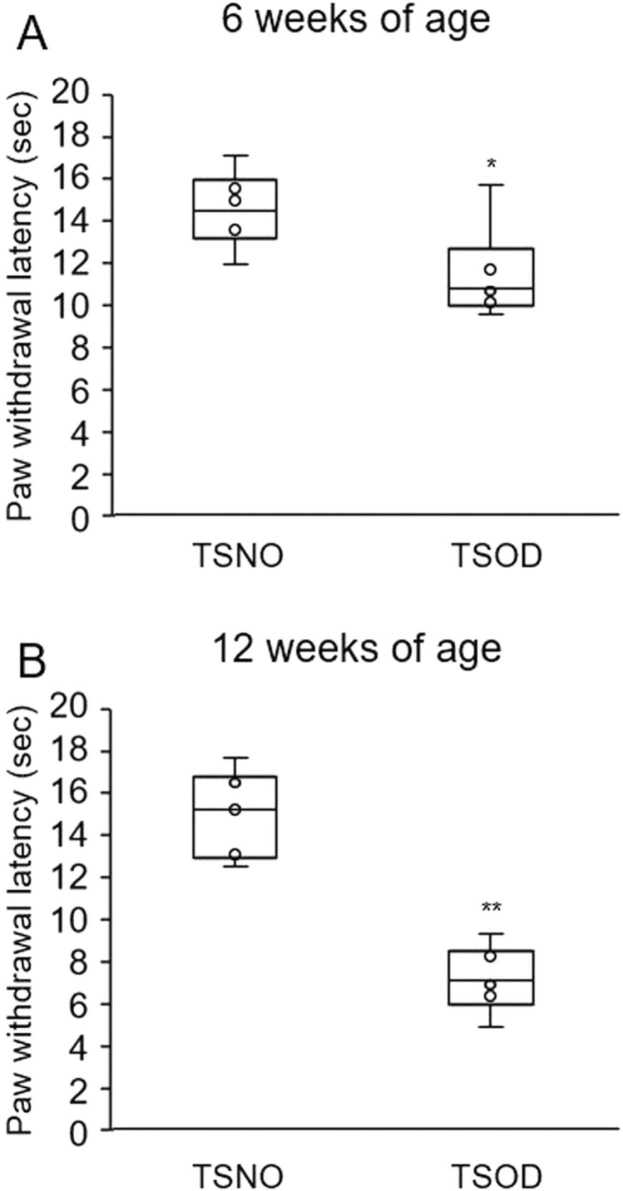
Changes in the thermal nociceptive threshold in 6-week-old (A) and 12-week-old (B) TSOD and TSNO mice. Mice were placed on a hot plate at 51 °C, and the latency to nociceptive response (licking hind legs or jumping) was measured. The cut-off time of 60 s was set to avoid tissue damage to the mice’s feet. Mice that did not respond within 60 s were removed from the apparatus and assigned a score of 60 s. Five measurements were taken per mouse, and the latency of the nociceptive response was determined by averaging the 3 measurements, excluding the maximum and minimum values. Data were presented as median and interquartile range (box and whisker) plus circles from 6 mice. Although only three points are visible in the graph, there are many close values, and they overlap. *P < 0.05, **P < 0.01; TSOD mice vs. TSOD mice.

To evaluate thermal hypersensitivity in TSNO and pre-DM state TSOD mice, the hot plate test was performed (n = 6). At 6 weeks of age, the mean response time to thermal stimulation was 11.6 ± 0.9 s in TSOD mice, which was significantly shorter than that in TSNO mice (14.6 ± 0.7 s, p < 0.05, Fig. 4A). At 12 weeks of age, mean reaction times were 15.2 ± 0.8 and 7.2 ± 0.6 s in TSNO and TSOD mice, respectively (p < 0.01, Fig. 4B), indicating a significantly shorter duration in TSOD mice.

### 3.5 In TSOD mice, the distribution of TRPV1-positive cells in dorsal root ganglia was shifted to larger myelinated cells

**Fig. 5:**
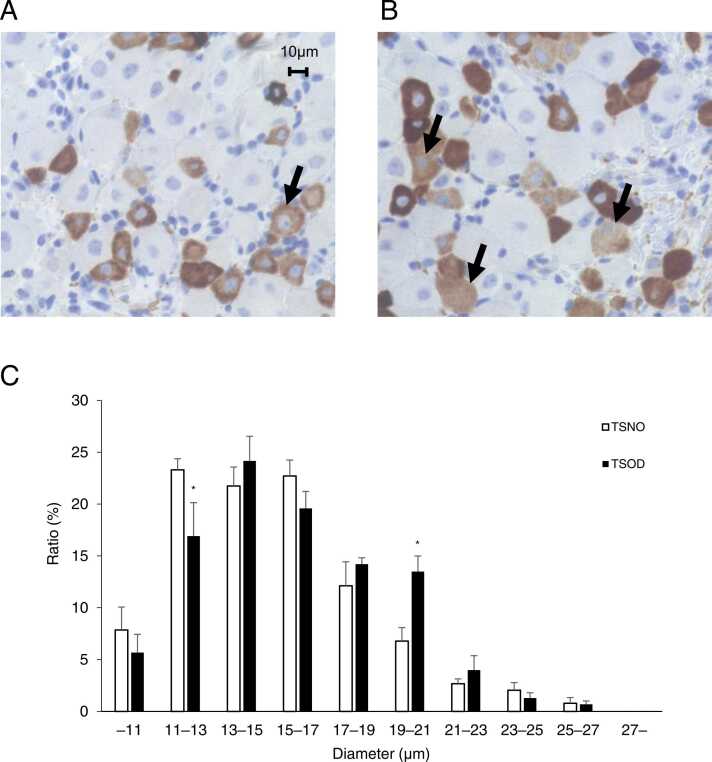
TRPV1 expression in the dorsal root ganglia (DRG) of TSOD and TSNO mice analyzed by immunohistochemistry. DRGs of 12-week-old TSNO and TSOD mice were collected and stained with a TRPV1 antibody. TRPV1-positive cells were stained in brown, and positive cells (arrows) larger than 10 µm were rarely observed in TSNO mice (A), but frequently observed in TSOD mice (B). The histogram of the diameter of neurons in the DRGs (C) clearly indicated that the percentage of cells with a diameter of 11–13 µm was significantly decreased and the percentage of cells with a diameter of 19–21 µm was significantly increased in TSOD mice compared to TSNO mice. Each bar represents the mean ± S.E.M. of 5 mice. Significant differences between groups were assessed by two-way ANOVA followed by the Tukey test. *P < 0.05, TSNO mice vs. TSOD mice. Scale bar = 10 µm.

To investigate the anatomical mechanisms of hyperalgesia in TSOD mice, which have a lowered thermal and mechanical nociceptive threshold in the pre-DM state, we evaluated the diameter of TRPV1-positive cells in DRG (Ohsawa et al., 2013). L4–6 DRG were removed from 12-week-old TSNO and TSOD mice, paraffin-embedded and sectioned, and immunostained with anti-TRPV1 antibody (n = 5). TRPV1-positive cells stained in light brown were mostly small cells with a diameter of about 10 µm and there were fewer large cells (Fig. 5A, arrows) in TSNO mice. In contrast with TSNO mice, TRPV1-positive cells in TSOD mice had fewer small cells with a diameter of about 10 µm and more large cells (Fig. 5B, arrows).

The diameter of TRPV1-positive cells was then assessed using ImageJ image analysis software, and a histogram was generated and evaluated (Fig. 5 C). The number of positive cells in a diameter of 11–13 µm was significantly lower in TSOD mice compared to that in TSNO mice (P < 0.05). The number of positive cells in the diameter of 19–21 µm was significantly higher in TSOD mice compared to that in TSNO mice (P < 0.05). The distribution of TRPV1-positive cells in TSOD mice was shifted from smaller cells to the larger cells.

**Fig. 6:**
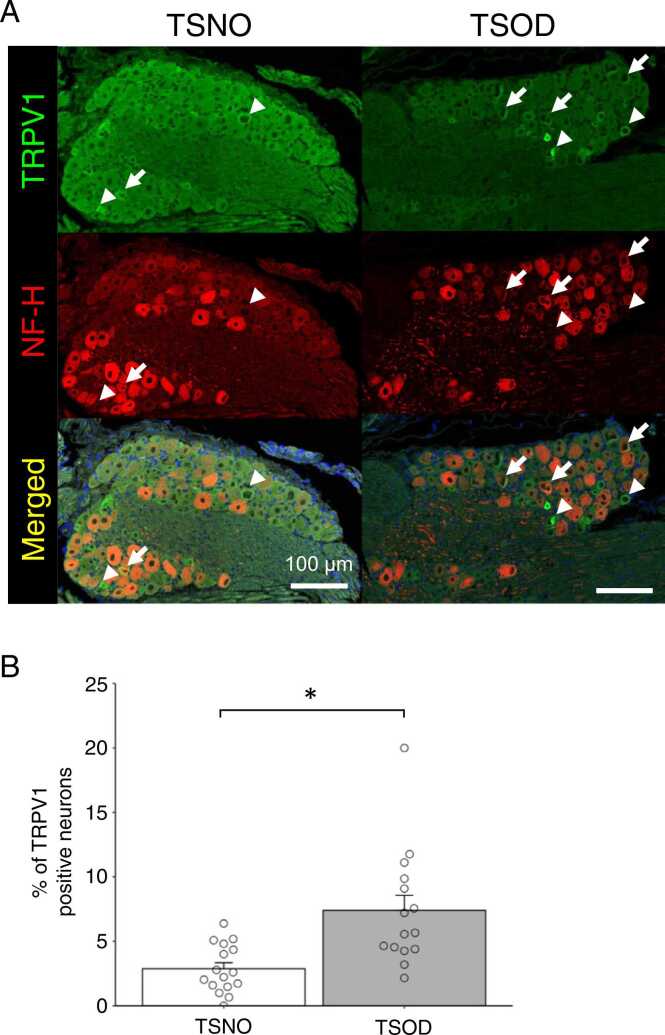
Double immunohistochemical staining of TRPV1 (green) and neurofilament heavy (NF-H; red) in lumbar DRG sections in TSNO (A, left) and TSOD (A, right) mice. Arrows and arrow heads indicate the myelinated and unmyelinated nerves expressing TRPV1, respectively. (B) The percentage of TRPV1-positive neurons that colocalized with NF-H in TSNO and TSOD mice. Each column represents the mean with S.E.M from 16 (TSNO, n = 5) and 15 (TSOD, n = 5) DRGs. The percentage of TRPV1-positive cells that colocalized with NF-H in TSOD mice was higher than that in TSNO mice (*P < 0.05, student’s t-test). Scale bar = 100 µm.

To determine the changes in TRPV1- immunoreactive (ir) expression in the DRG of TSOD mice, double fluorescence labeling was conducted in TSNO and TSOD mice. We used a myelinated neural marker, NF-H, to distinguish the subgroup of DRG neurons. The majority of NF-H-positive neurons lacked TRPV1-ir in the DRG of TSNO mice (Fig. 6A, left). In TSOD mice, TRPV1-ir was expressed in NF-H-positive neurons (Fig. 6A, right). The population of TRPV1-expressing myelinated nerves in the DRG in of TSOD mice was significantly higher than that of TSNO mice (Fig. 6B).

## 4 Discussion

In this study, we detected IGT neuropathy-like symptoms in the pre-DM state of TSOD mice, a model of type 2 DM. We also found that TRPV1-expressing cells in the DRG were larger in pre-DM state TSOD mice than they were in TSNO mice. TRPV1 expression in larger myelinated cells changed sensory information into nociception and lowered the thermal and mechanical nociceptive threshold.

TSOD mouse is a spontaneous model of type 2 DM that was generated from ddY mice in 1992. It is a multifactorial genetic model (with mutations on chromosomes 1, 2, and 11 (Hirayama et al., 1999)) and exhibits visceral obesity. Since these features are similar to those seen in the obese Asian population, it is a good model for metabolic syndrome research.

We first investigated several parameters related to diabetes in TSOD mice aged 5–16 weeks. TSOD mice showed significantly heavier body weight than age-matched non-diabetic TSNO mice (Fig. 1 A). Interestingly, the non-fasting blood glucose levels up to 12 weeks of age were not elevated in TSOD mice. Non-fasting blood glucose levels at 13 weeks were slightly elevated in TSOD mice compared to those in TSNO mice (Fig. 1B). Urine glucose positivity in TSOD mice increased rapidly after 13 weeks (Fig. 1C). Few previous studies have examined these parameters in TSOD mice weekly from a young age. However, non-fasting blood glucose levels in TSOD mice were reported to be below 250 mg/dL from 4 to 13 weeks of age and to increase after 14 weeks of age (Murotomi et al., 2015). The results of this study follow previous results, but there is a gap in the timing of the increase in blood glucose levels. It would be possible for TSOD mice to be multifactorially inherited, and for environmental factors to affect the diabetes onset time.

Many reports have investigated glucose tolerance in TSOD mice. The glucose tolerance abnormalities in TSOD mice have been reported at 8, 11, 13, 15, and 24 weeks of age (Murotomi et al., 2015), and 20 weeks of age (Shimada et al., 2014). In this study, the ITT was performed in 5-week-old TSNO and TSOD mice, and insulin resistance was observed in TSOD mice (Fig. 2).

Based on these results, we hypothesized that the “pre-DM state of TSOD mice” would be up to 12 weeks of age, when the mean non-fasting blood glucose levels are less than 250 mL/dL, urine glucose positivity is less than 40%, and insulin resistance is present. The American Diabetes Association classification defines the human pre-DM state as HbA1c = 5.7–6.4% (Stino and Smith, 2017). In humans, HbA1c reflects changes in blood glucose levels from one to two months before. In mice, it reflects past serum glucose levels, although the timing is different as the lifespan of red blood cells is different from that of humans. On the other hand, the non-fasting blood glucose levels are a guide for understanding the current blood glucose level, which rises with meals. In this study, we measured non-fasting blood glucose instead of HbA1c, because our experiment evaluated the mechanical nociception threshold in the IGT state, and we thought that the measurement of serum glucose levels would be sufficient and convenient to confirm the IGT state. Therefore, the results of this study may not be completely consistent with the pre-DM state in humans.

In pre-DM state TSOD mice, the von Frey test showed a significant decrease in the mechanical nociceptive threshold (Fig. 3). Since the decrease in nociceptive threshold for mechanical stimulation was confirmed from 5-weeks of age in TSOD mice, it is possible that these mice have a naturally lowered mechanical nociceptive threshold. However, TSOD mice have been reported to be under oxidative stress from the age of 5 weeks (Murotomi et al., 2014). Indeed, 5-week-old TSOD mice showed no increase in blood cytokines (TNF-α and IL-6), but showed an increase in the oxidative stress marker plasma tHODE (total hydroxyoctadecadienoic acid). This suggests that TSOD mice develop DM when exposed to higher oxidative stress at a young age, and the lowered nociceptive threshold in TSOD mice is also caused by oxidative stress.

In this study, we found that thermal and mechanical nociceptive thresholds were decreased in insulin-resistant mice. Insulin plays key roles in the central and peripheral nervous systems, such as promoting nerve regeneration inducing neurite outgrowth, maintaining mitochondrial function, supporting memory formation, and regulating hypothalamic metabolism. Although altered insulin signaling is a major factor in the development of DM, its role in DM neuropathy is not well understood. However, it has been reported that low doses of insulin can improve the symptoms of neuropathy without affecting blood glucose levels (Sugimoto et al., 2013), suggesting that insulin has a beneficial effect on neuropathy.

Furthermore, insulin resistance has also been found in vivo in the peripheral nerves of ob/ob mice with DM peripheral neuropathy, the same type 2 DM model as TSOD mice. It has been suggested that altered insulin signaling in the peripheral nerves contributes to DM neuropathy (Grote et al., 2013). In this paper, Grote et al. showed that insulin-induced activation of Akt, a downstream signaling molecule of the insulin receptor, is reduced in the DRG and sciatic nerve of insulin-resistant ob/ob mice. Similar changes in insulin signaling in the peripheral nerves may be involved in peripheral neuropathy in this study.

We confirmed the shortening of the duration of nociceptive response to thermal stimuli in the hot plate test in pre-DM state TSOD mice at 6 and 12 weeks of age (Fig. 4A–B). Since the shortened response time to thermal stimulation in TSOD mice was also observed at 6 weeks of age, we cannot deny the possibility of it being a natural characteristic of TSOD mice. In a previous study, we reported that LPA (lysophosphatidic acid) induced mechanical allodynia without affecting the thermal nociceptive sensitivity (Ohsawa et al., 2013). It is thought that hypersensitivity to thermal stimulation did not appear after LPA treatment, because there was demyelination of the sensory nerves. Since TSOD mice are hypersensitive to thermal and mechanical stimulation, the demyelination of sensory nerves may not be involved in TSOD mice neuropathy.

In addition to TSOD mice, DM models such as streptozotocin (STZ) -induced DM mice (Watcho et al., 2011), Akita mice (Drel et al., 2011), and high-fat diet (HFD) mice (Obrosova et al., 2007), also show reduced mechanical nociceptive thresholds and shorter response times to thermal stimuli. In a mouse model of HFD, mechanical allodynia and thermal hypersensitivity improve after 6 weeks of treatment with a normal solid diet. Therefore, the lowered mechanical nociceptive threshold and shortened reaction time to thermal stimuli in the pre-DM state are thought to be due to functional abnormalities of the peripheral nerves and not to structural abnormalities (Obrosova et al., 2007). In the same way, IGT neuropathy in TSOD mice could be improved with intervention.

To investigate the mechanism of hypersensitivity to mechanical and thermal stimuli in pre-DM state TSOD mice, we measured the diameter of TRPV1-positive cells in the DRG, a region closely associated with pain (Hong et al., 2008, Ohsawa et al., 2013, Pabbidi et al., 2008). The results showed a decrease in the percentage of small TRPV1-positive cells, and an increase in the percentage of large cells in the DRG of TSOD mice (Fig. 5 C). The DRG contain cell bodies of sensory nerves, with the larger ones (diameter > 35 µm) being cell bodies of Aβ fibers, which are myelinated nerves, and the smaller ones (diameter < 25 µm) being cell bodies of Aδ nerves, which have a thin myelin sheath, and C fibers, which are unmyelinated nerves (Lawson, 2002). Neurons in the DRG are an important target in neurological complications and are disrupted in DM neuropathy (Hong et al., 2008). TRPV1, a member of the vanilloid subfamily, is a Ca^2+^-permeable, non-selective cation channel that is activated by high temperature (<43 °C), low pH (<5.9), and oxidative stress of capsaicin and pain. Repeated activation of TRPV1 receptors results in the overload of intracellular Ca^2+^, leading to oxidative stress and neuronal apoptosis (Hong et al., 2008). In LPA-induced mouse models (Ohsawa et al., 2013) and STZ-induced diabetic mouse models (Hong et al., 2008), TRPV1 was also expressed in large cells of the DRG. The DRG in Akita mice, an animal model of type 1 DM (Chen et al., 2019), also showed increased TRPV1-positive cells. This is consistent with our results.

TRPV1 is mainly expressed in small diameter cells of DRG under normal conditions but was abundant in large cells in 12-week-old TSOD mice. We also performed double fluorescence labeling with NF-H, markers of myelinated nerves, and TRPV1, and found that TRPV1 expression was significantly increased in myelinated neurons of the DRG in 12-week-old TSOD mice. These results indicated that the TRPV1 expression pattern in the DRG changed in TSOD mice, suggesting that behavioral changes in TSOD mice are caused by the increased expression of TRPV1 in myelinated nerves. The results of present and previous studies suggest that the expression of TRPV1 in myelinated Aβ fibers has decreased the mechanical and thermal nociceptive threshold because of the transmission of tactile sensations into nociceptive sensations. However, it is also possible that TRPV1 expression is shifted to large diameter cells for lowering the nociceptive threshold.

In this study, we confirmed the pre-DM state in 5–12 weeks old TSOD mice. Additionally, we observed a lowered mechanical and thermal nociceptive threshold in these mice. These results suggest that TSOD mice can be a useful model for IGT neuropathy. We also suspected that the change in the TRPV1 expression pattern from small unmyelinated neurons to large myelinated neurons of the DRG may be involved in the pathogenesis of IGT neuropathy.

## Funding source

This research did not receive any specific grant from funding agencies in the public, commercial, or not-for-profit sectors.

## CRediT authorship contribution statement

**Kyoko Shida:** Performed the experiments, Collected and analyzed data, Was a major contributor in writing the manuscript. **Masahiro Ohsawa:** Designed and supervised this study and analyzed data. **Satoru Takahashi:** Supervised immunohistochemistry. **Haruko Ota, Tetsuya Tamura, Nobuyoshi Kusama:** Contributed to the experimental design and manuscript preparation. **Mina Nakasone, Hisaaki Yamazaki:** Immunohistochemistry data acquisition and analysis. **Kazuya Sobue:** Designed and supervised this study. All authors read and approved the final manuscript.

## Declarations of interest

None.
